# Significantly Improving the Power Capability of Water-Jet Guided Laser: An Optical Breakdown Suppression Strategy via Axial Multi-Focal Beam Shaping

**DOI:** 10.3390/mi17091071

**Published:** 2026-09-09

**Authors:** Dandan Zhao, Yugang Zhao

**Affiliations:** School of Mechanical Engineering, Shandong University of Technology, No. 266 Xincun West Road, Zibo 255000, China; zhaodandansdut@126.com

**Keywords:** water-jet guided laser, aspheric lens, beam shaping, high laser power, NiTi alloy

## Abstract

Water-jet guided laser (WJGL) technology has gained significant attention in precision manufacturing due to its extremely small heat-affected zone. However, laser-induced water breakdown severely constrains the achievable laser power and processing efficiency. This paper presents and validates an optical solution employing a custom-designed rotationally symmetric aspheric lens. The lens is designed to generate a sequence of discrete focal points distributed along the optical axis. This configuration maintains a high average laser power while suppressing the peak power density at each individual focus below the water breakdown threshold. Theoretical modeling and ray tracing simulations confirm the superior performance of the lens in creating a controllable multi-focal beam. Experimental results demonstrate that a WJGL system incorporating the six-focus aspheric lens operates stably at 350 W. This represents a 300 W increase compared to the conventional spherical lens, which had a stable operating power limit of approximately 50 W within this experimental system. In microgroove machining experiments on NiTi alloy, the new system achieved an approximately 3.5-fold increase in groove depth and a 2.7-fold reduction in taper angle. This study provides a practical and effective beam shaping strategy to overcome the fundamental power limitation in WJGL technology.

## 1. Introduction

Water-jet guided laser (WJGL) machining is a non-contact precision technology that employs a stable water jet to guide a high-energy laser beam onto the workpiece. Capitalizing on the continuous cooling and efficient removal of molten debris by the water jet, this technique significantly mitigates thermally induced defects such as heat-affected zone, micro-cracks, and recast layers [1,2]. Consequently, it is particularly well-suited for machining thin-walled components [3], brittle materials [4], and complex three-dimensional structures [1]. The technology has been successfully applied in high-end manufacturing, including the machining of aero-engine film cooling holes [5], semiconductor wafers [6], ultra-hard materials [7], and composite materials [8].

Despite these advantages, its industrial adoption suffers from low machining efficiency. The primary cause of this limitation is the stringent power threshold imposed by laser-induced water breakdown [9,10,11]. When the laser power exceeds a critical threshold, optical breakdown is triggered in the water jet [12], causing water jet turbulence and disrupting energy transmission. Furthermore, the coupling of high-power lasers (particularly in the infrared spectrum) with the water jet leads to localized heating due to water’s absorption of optical energy [13], thereby inducing water cavitation. The extreme mechanical loads generated upon cavitation bubble collapse—with shock wave pressures reaching the GPa level—can damage the nozzle [14,15,16]. More critically, when the laser power density surpasses the water breakdown threshold (approximately 10^10^ W/cm^2^ for 1064 nm nanosecond lasers), avalanche ionization is initiated, generating plasma and disrupting the total internal reflection condition of the laser within the water jet [17,18]. This physical constraint strictly limits the maximum usable power in WJGL systems. For instance, recent studies typically report maximum laser powers below 300 W [5,19,20,21], which severely restrict the technology’s application in efficient cutting, particularly for thick-walled components [21].

Therefore, overcoming the power limitation caused by optical breakdown is the key challenge for advancing this technology. Conventional optical systems using spherical lenses inherently concentrate laser energy at a single focal point, easily triggering optical breakdown [8,22,23]. Simply increasing laser power leads to severe instability, as evidenced by the flashes and explosions observed by Liu et al. [24], as well as the potential nozzle damage due to nonlinear absorption of the high-power laser [25]. Energy losses during high-power coupling—approximately 30% irradiance loss, as reported by Spiegel [13]—further exacerbate the problem. Thus, the key to resolving the power limitation lies in effectively reducing the peak power density in the focal region while maintaining a high average laser power.

Beam shaping technology offers a potential pathway to overcome this challenge by reshaping the energy distribution of the optical field to optimize machining outcomes [26]. The design of the focusing lens is critical, as it directly determines the energy distribution at the focal spot. With the maturation of technologies such as magnetorheological finishing (MRF) [27,28], aspheric lenses with complex profiles can now be fabricated cost-effectively and with high precision, enabling their use in optical system performance optimization [29,30,31]. Research demonstrates that aspheric lenses can not only simplify system architecture [32] but also effectively transform Gaussian beams into top-hat beams [33,34] or meet specific illumination requirements [35]. Recent work highlights their potential in high-end manufacturing; for example, randomizing the focal spot array to improve energy uniformity [36]. Furthermore, researchers have proposed adaptive aspheric beam shaping systems that achieved a 70% improvement in microstructure machining efficiency [37]. Therefore, research into optimizing spot quality to enhance the coupling power and efficiency of water-jet guided lasers has gradually attracted increasing attention. In 2022, Huang et al. [38] combined an annularly bonded concave axicon lens with a convex axicon lens. By modulating the phase distribution of the laser beam, a non-diffracting beam was generated, achieving high tolerance to random coupling along the z-axis and thereby improving coupling efficiency. In 2023, Wang et al. [39] controlled the beam quality by adjusting the resonator length and mirror curvature to enhance the transmission efficiency of water-jet guided lasers, successfully increasing and stabilizing the transmission efficiency in the range of 88.2% to 90.5%. In 2025, Wang et al. adopted the dual-beam water jet laser (DWJL) technique, by which two laser beams were effectively coupled into a 100 μm diameter water jet, raising the maximum pulse energy to 4.63 mJ [40] and substantially improving the processing efficiency of water-jet guided laser processing. Although the aforementioned studies have enhanced the processing capabilities of water-jet guided laser technology through optical design, seeking a simpler and more effective method to improve its processing efficiency remains a critical area that requires further investigation.

This study systematically introduces the concept of axial multi-focal beam shaping to water-jet guided laser (WJGL). We designed a rotationally symmetric aspheric lens with continuously varying curvature. This lens transforms the incident collimated beam into a series of discrete focal points distributed along the water jet axis. The core physical principle involves the spatial redistribution of the total laser energy along the beam path. This approach maintains a high average laser power while ensuring that the peak power density at each focal point remains below the water breakdown threshold. Through theoretical analysis, optical design, numerical simulation, and experimental validation, this paper demonstrates the effectiveness and superiority of this strategy in increasing power handling capability, suppressing optical breakdown, and improving machining efficiency.

## 2. Laser-Induced Water Breakdown

### 2.1. Breakdown Phenomena and Damage Characterization

**Figure 1:**
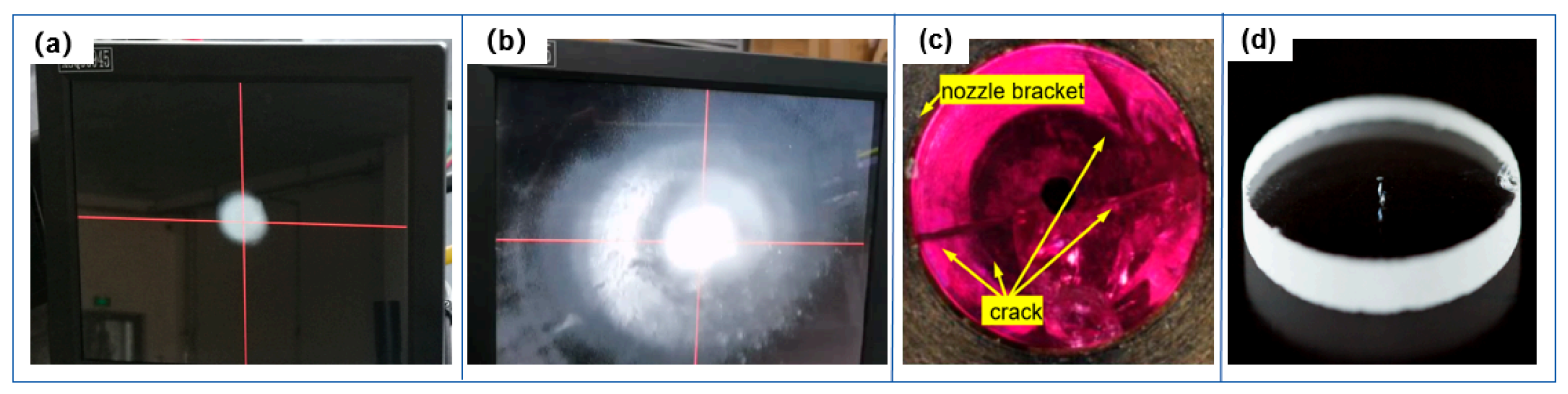
Laser-water jet coupling monitoring images and nozzle damage morphology: (**a**) normal state; (**b**) plasma flash induced by optical breakdown; (**c**) nozzle fracture; (**d**) ablation mark on the optical window.

The key parameters of the water-jet guided laser experimental system used in this study are presented in Table 1. Experiments were conducted under the following conditions: laser wavelength 1064 nm, laser pulse frequency 5 kHz, pulse duration 500 ns (SQ mode), working medium deionized water, nozzle diameter 0.5 mm, focal length 100 mm, and spot diameter 18 µm. During preliminary tests with an average laser power of 120 W using the water-jet guided laser system with a single-focus lens, the water jet was found to be extremely unstable, accompanied by a loud explosive sound. A CCD camera captured an intense plasma flash at the laser focus (Figure 1b), in contrast to the normal machining condition (Figure 1a). Subsequent disassembly revealed that the nozzle exhibited a characteristic brittle fracture morphology (Figure 1c). Simultaneously, a distinct ablation mark was observed at the center of the coupling chamber’s optical window (Figure 1d). This evidence confirms that the extreme energy released during laser-induced water breakdown caused substantial damage to core components.

### 2.2. Physical Mechanism of Laser-Induced Breakdown

The nonlinear interaction between the laser and the water medium is the primary cause of breakdown. The process can be understood through three interrelated mechanisms.

Plasma Formation and Shock Wave Excitation. When the local laser power density exceeds the breakdown threshold of water, the water in the focal region is instantaneously ionized, forming a high-temperature, high-pressure plasma. The explosive expansion of this plasma generates an intense shock wave in the water, which reaches an extremely high sound pressure level (>260 dB) [41]—a phenomenon known as the “laser-induced acoustic effect.” Consequently, the explosive sound observed experimentally originates primarily from this shock wave [42,43,44].

Optocavitation Effect and Mechanical Damage. Concurrent with plasma formation, vigorous optocavitation is induced [45]. The cavitation bubbles generated can collapse near rigid boundaries such as the nozzle wall, producing high-speed microjets and secondary shock waves with pressures reaching the GPa level [46]. Their instantaneous load far exceeds the yield strength of the nozzle material, leading to irreversible damage.

Ionization Mechanism. For 1064 nm nanosecond lasers, water breakdown is dominated by avalanche ionization. Seed electrons absorb laser energy via inverse bremsstrahlung, triggering an avalanche-like growth in electron density until water breakdown occurs [47,48]. This is precisely the direct cause of nozzle rupture. Therefore, it is imperative to introduce the classical avalanche ionization model and quantitatively calculate the theoretical breakdown threshold under the experimental conditions of this study.

### 2.3. Threshold Theory and Multi-Focal Suppression Mechanism

In 1995, Kennedy derived and established the classical avalanche ionization model (see Ref. [49], Equations (58) and (59)) and investigated the breakdown behavior of nanosecond laser pulses in pure water at wavelengths of 532 nm and 1064 nm. Based on this model, Noack et al. [50] studied the calculation methods for the threshold, absorption coefficient, and energy density of laser-induced plasma formation in water across timescales ranging from nanoseconds to femtoseconds. Experimental studies based on these findings indicate that the breakdown threshold for nanosecond laser pulses at a wavelength of 1064 nm in pure water is approximately 10^10^ W/cm^2^ [12,51,52].

The breakdown threshold can be described by the following equation [49]:

(1) $$I_{\mathrm{th}}\;=\;\frac{C_{1}\left(1\;+\;\omega^{2}\tau^{2}\right)}{\tau }\left[g\;+\;\frac{2}{\tau_{p}}\ln\left(\frac{\rho_{\mathrm{cr}}}{\rho_{0}}\right)\right]\;+\;\frac{C_{1}m\omega^{2}}{M}$$

where $C_{1}\;=\;\frac{\mathrm{mc}n_{0}\epsilon_{0}E_{\mathrm{ion}}}{e^{2}}$; m is the electron mass, c is the speed of light in vacuum, n_0_ is the refractive index of water, ϵ_0_ is the vacuum permittivity, E_ion_ is the ionization energy of water molecules, ω is the angular frequency of the laser, τ is the electron collision mean free time, e is the elementary charge, g is the electron loss rate, τ_p_ is the laser pulse width, ρ_cr_ is the critical plasma density, ρ_0_ is the initial electron density, and M is the mass of a water molecule.

Experimental parameters for traditional single-focus lenses (average laser power 120 W: the laser pulse duration is 500 ns (SQ mode), spot diameter 18 μm), the single-pulse energy E_p_ = P_avg_/ν is 24 mJ. The single-pulse peak power density, calculated as I_p_ = 4 × E_p_/(πd^2^τ_p_), is approximately 1.89 × 10^10^ W/cm^2^. This value is comparable to or slightly exceeds the theoretical breakdown threshold of approximately 10^10^ W/cm^2^, theoretically confirming the occurrence of breakdown. Conversely, it can be deduced that in the current system, an average power of approximately 63.6 W (theoretical value) is sufficient for the peak power density to reach the breakdown threshold.

Multi-focal System Suppression Mechanism. Based on the above threshold analysis, a multi-focal strategy can be employed to suppress breakdown by distributing the total power across multiple foci. In contrast to the traditional single-focus system, the multi-focal beam distributes the total energy Ptotal among N effective focal points. If the effective spot area at each focus is Aspot, then the average power density at each focus is

I_single_ ≈ P_total_/(N × A_spot_) (2)

By ensuring I_single_ < I_threshold_ (the breakdown threshold) through design, plasma formation can be fundamentally suppressed, thereby enabling stable coupling at high total power. That is, in an axial multi-focus beam shaping system, when the power density at each focal spot is ensured to be below the water breakdown threshold, each focal spot carries 1/N of the total laser power. Therefore, such a system can increase the total laser power while avoiding nozzle damage that would otherwise occur during high power laser-water jet coupling.

## 3. Design Principle of Aspheric Lens

### 3.1. Optical Design Principle

**Figure 2:**
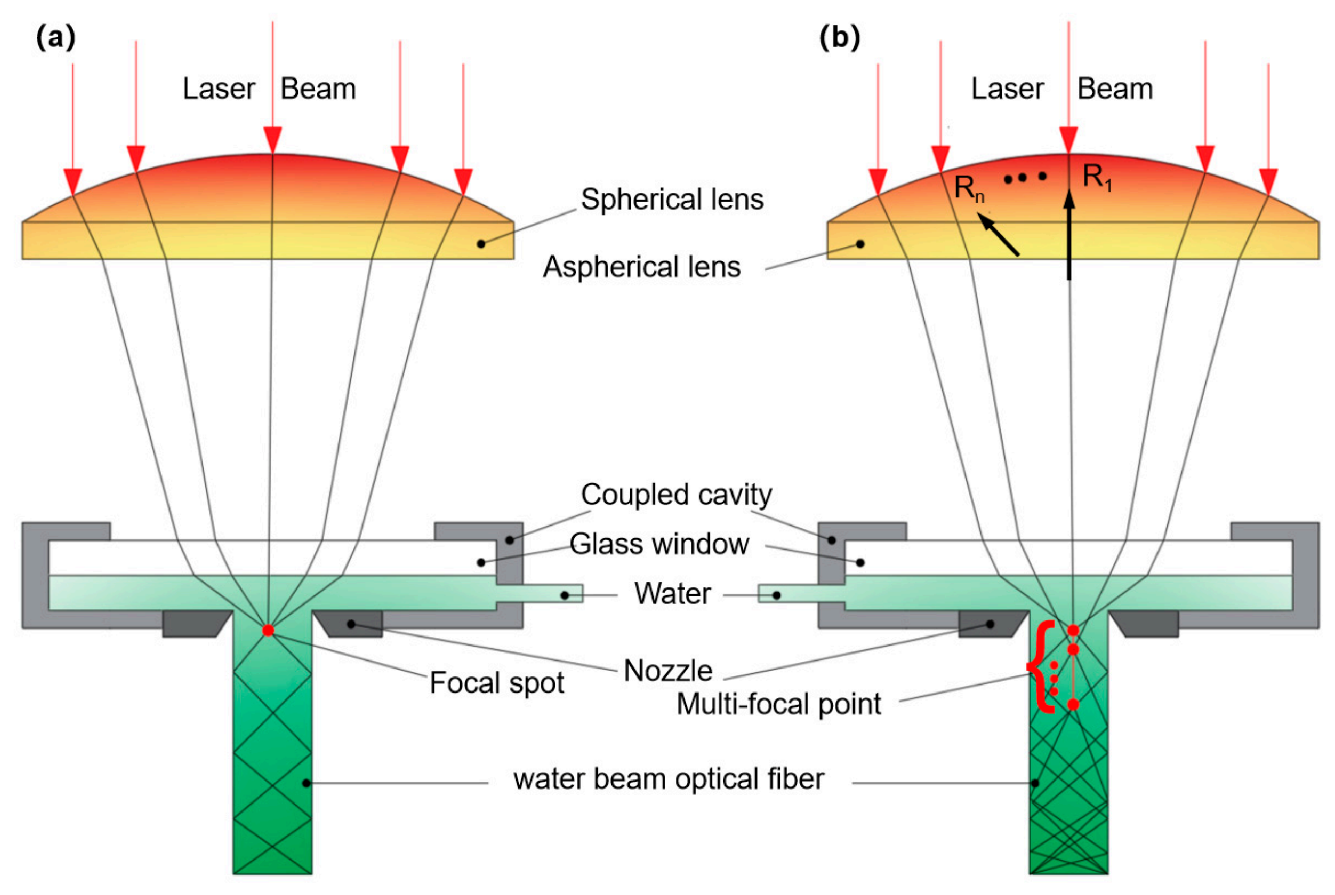
Schematic diagram of laser–water jet coupling principle: (**a**) laser–water jet coupling based on spherical lens; (**b**) laser–water jet coupling based on aspheric lens.

Figure 2 illustrates the operating principles of laser–water jet coupling using a spherical lens (Figure 2a) and an aspheric lens (Figure 2b). When a collimated laser beam is incident on the aspheric lens, its curvature varies continuously with radial position. Consequently, rays at different radial coordinates are refracted by varying angles, converging into a predefined sequence of focal points along the central axis of the water jet.

To achieve this functionality, the contour curve Z(r) of the lens front surface requires precise definition. The front surface is described using the rotationally symmetric even-order aspheric equation, which offers excellent fitting convergence and is widely used in optical design [53]:

(3) $$Z\;(r)\;=\;\frac{cr^{2}}{1\;+\;\sqrt{1\;-\;\left(1\;+\;k\right)c^{2}r^{2}}}\;+\;{\;A}_{4}r^{4}\;+\;{\;A}_{6}r^{6}\;+\;A_{8}r^{8}\;+\;\cdots$$

where Z is the sagitta (surface height), r is the radial coordinate, c is the vertex curvature, k is the conic constant (k = −e^2^), and A_4_, A_6_, A_8_, … are aspheric coefficients.

### 3.2. Surface Model and Calculation Method

The modeling is based on three key assumptions: (1) refraction occurs once at the lens front aspheric surface and the rear planar surface; (2) the lens material is a homogeneous isotropic medium with refractive index n (relative to vacuum); (3) the surrounding medium is air, with refractive index n_2_ ≈ 1. The system is rotational symmetric, enabling the calculation of the cross-sectional profile in the meridional plane.

**Figure 3:**
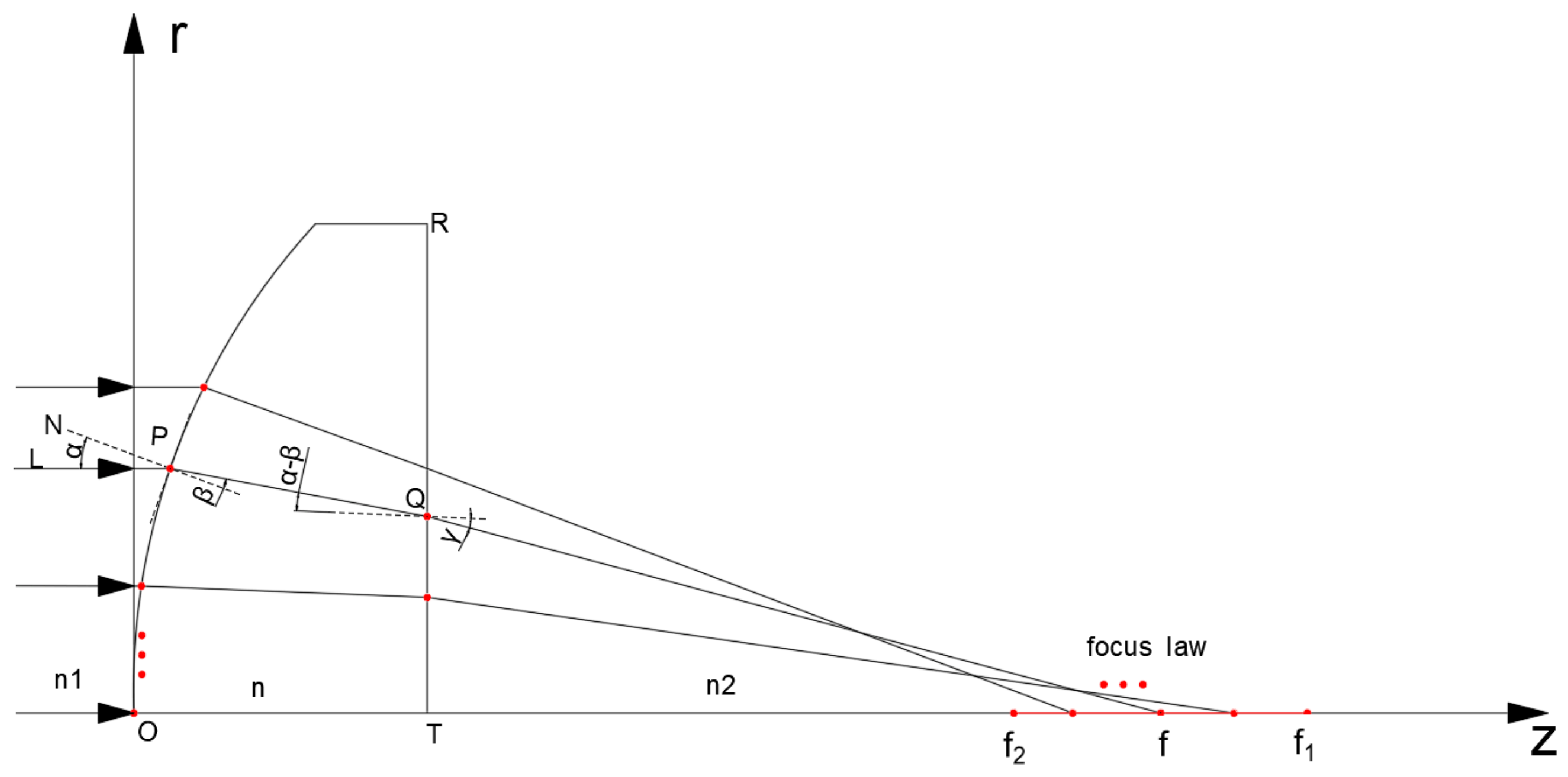
Surface model and focusing characteristics of the aspheric lens.

For a collimated incident beam, the optical path is illustrated in Figure 3. Based on Snell’s law and geometric optics ray tracing equations, OT is the maximum thickness of the lens and assuming tanα = k for any point P(z_i_,r_i_) on the lens surface with refractive index n, the expression for the z-axis coordinate z of focus F can be derived as Equation (4). The detailed derivation is provided in the Appendix A.

(4) $$\tilde{z}=\mathrm{OT}-\left[r_{i}+\frac{\left(\sqrt{n^{2}k^{2}+n^{2}-k^{2}\;}-1\right)k}{\sqrt{n^{2}k^{2}+n^{2}-k^{2}}+k^{2}}\left(\mathrm{OT}-z_{i}\right)\right]\;\times \;\frac{\sqrt{\left(2-n^{2}\right)\;k^{4}+\left(1-n^{2}\right)\;k^{2}+2k^{2}\sqrt{n^{2}k^{2}+n^{2}-{\;k}^{2}}+1}}{\left(\sqrt{n^{2}k^{2}+n^{2}-k^{2}}-1\right)k}$$

**Figure 4:**
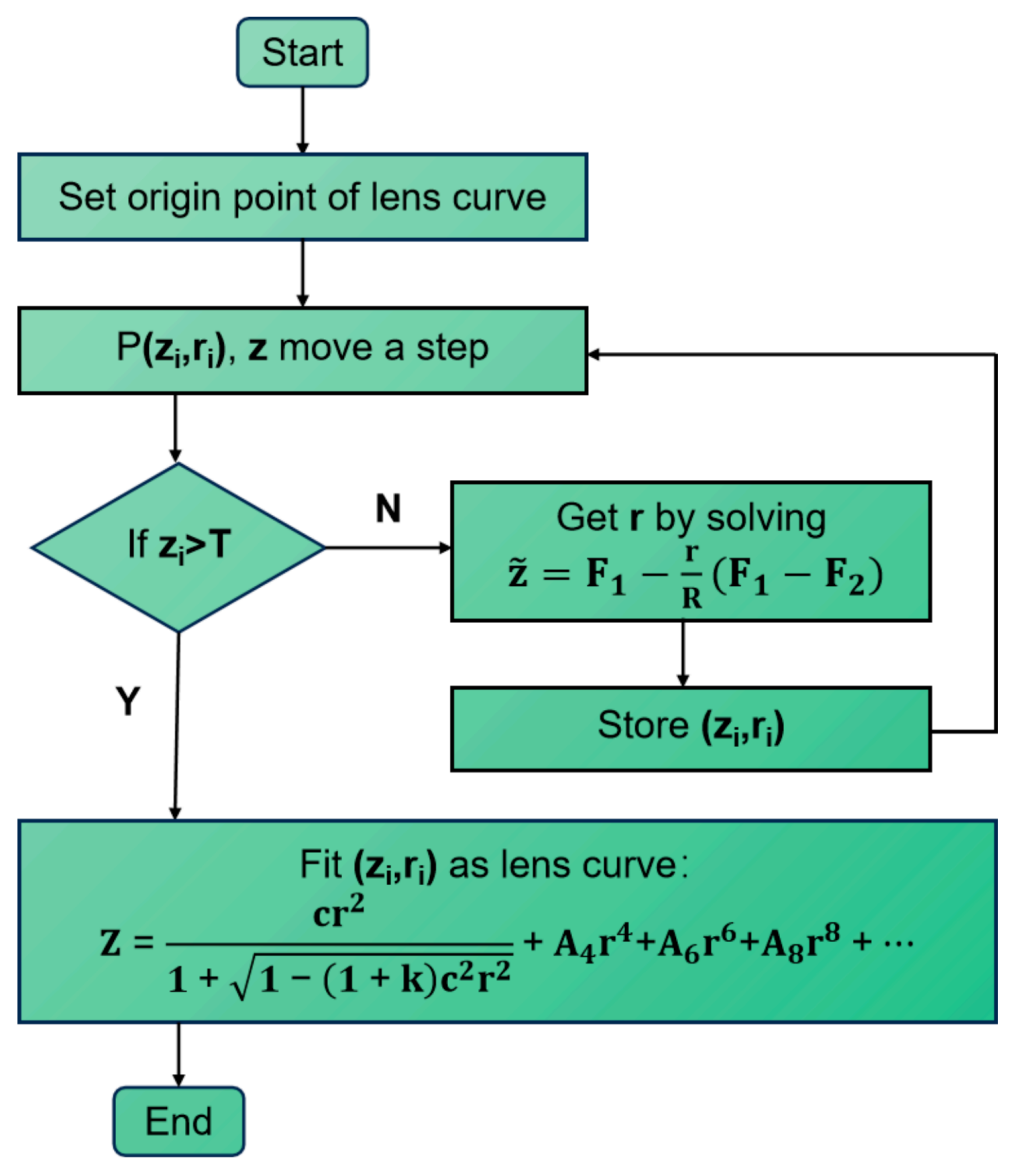
MATLAB programming flowchart.

Equation (4) can be simplified to:

(5) $$\tilde{z}\;=\;f_{1}\;-\;\frac{r_{i}}{R}\left(f_{1}\;-\;f_{2}\right)$$

where f_1_ and f_2_ are focal position parameters, r_i_ is the radial coordinate, and R is the rear surface radius. The detailed derivation process of Equation (4) can be found in the Appendix A. Subsequently, specific parameters are assigned based on the lens application conditions, including the focal range f_1_–f_2_, the z-axis coordinate z of any desired focus F, and the lens rear surface radius R. A MATLAB (R2020a) program (Figure 4) solves Equation (5) to obtain the point set {P(z_i_, r_i_)}. Finally, this point set defining the lens surface is substituted into Equation (3) to determine the aspheric coefficients, completing the lens design.

To ensure stable laser transmission via total internal reflection within the water jet, this condition must be satisfied. The critical angle θ_c_ for total internal reflection at the water (optically denser medium, n_1_ ≈ 1.33) to air (optically less dense medium, n_2_ ≈ 1) interface is given by Snell’s law:

(6) $$\theta_{c}\;=\;\arcsin\left(\frac{n_{2}}{n_{1}}\right)$$

substituting the values into Equation (6) yields θ_c_ ≈ 48.75°. Therefore, total internal reflection occurs when the laser incidence angle is ≥48.75°. This provides the theoretical foundation for the subsequent multi-focal aspheric lens design.

## 4. Numerical Simulation and Results Analysis

**Figure 5:**
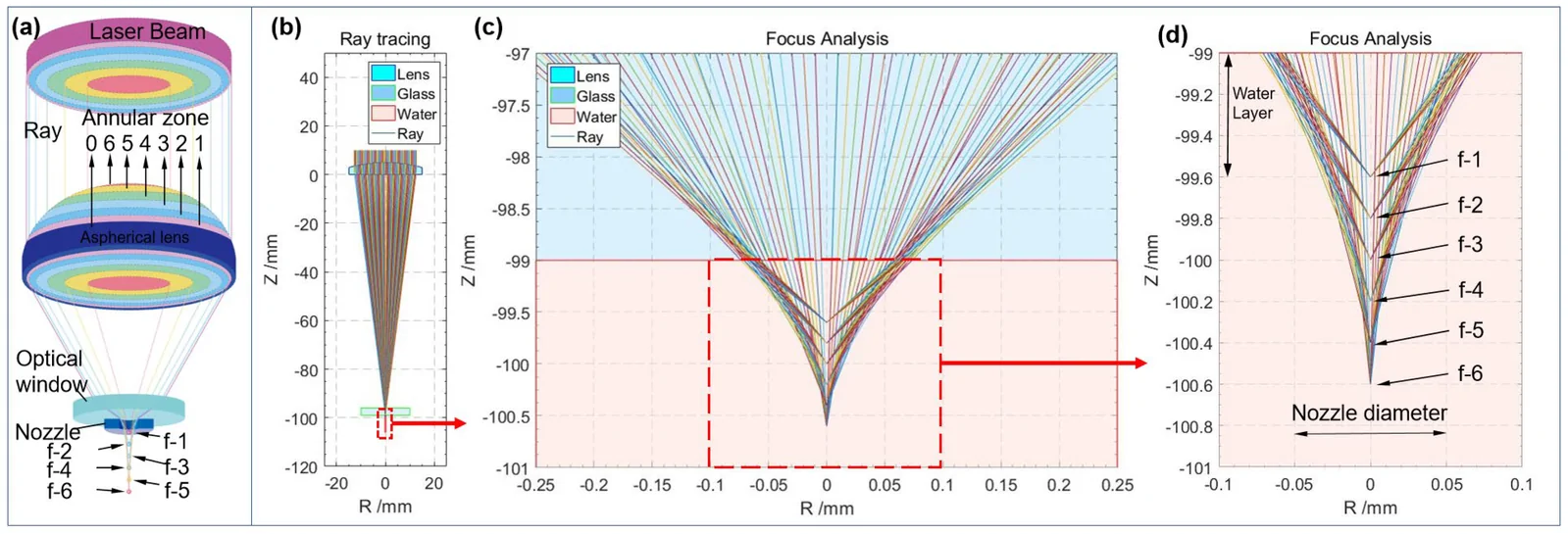
Focusing model and simulated ray tracing results of the six-focus aspheric lens: (**a**) schematic diagram of the focusing model; (**b**) simulated ray tracing results of the six-focus aspheric lens; (**c**) ray tracing results in the optical window and water medium; (**d**) ray tracing results in the water medium, showing six focal lengths of 99.6 mm, 99.8 mm, 100 mm, 100.2 mm, 100.4 mm, and 100.6 mm, with an axial spacing of 0.2 mm between adjacent foci.

To validate the optical performance of the aspheric lens designed in Section 3, a six-focus aspheric lens was selected as an application example based on the physical constraints of the experimental platform and the feasibility of lens fabrication. Ray tracing simulations were conducted using MATLAB, and the specific simulation workflow and key parameters are described as follows:

First, the optical system parameters were defined based on the physical constraints of the water-guided laser platform, as listed in the left column of Table 2. These include lens geometry, material properties (BK7 glass, n = 1.5168 at 1064 nm), and performance targets. The surface parameters of the aspheric lens were determined using the mathematical model from Section 3. The resulting design parameters for the six-focal-point aspheric lens are listed in the right column of Table 2. The surface of the six-focus aspheric lens is defined by the even-order aspheric equation (Equation (7)). This design modulates the numerical aperture into different annular zones (Figure 5a), thereby creating a multi-focal characteristic.

(7) $$Z(r)=\frac{0.0195r^{2}}{1+\sqrt{1-0.0195^{2}r^{2}}}+3.42\;\times \;10^{-6}r^{4}-2.58\;\times \;10^{-8}r^{6}+4.88{\;\times {\;10}^{-10}r}^{8}-1.89\;\times {\;10}^{-12}r^{10}$$

A 2D axisymmetric model was then established based on Equation (7) to accurately represent the rotationally symmetric lens structure. The model incorporated the refractive indices of air, the lens material (BK7), the glass window, and water at the 1064 nm laser wavelength. This model provided the ray propagation trajectories and focal point distribution (Figure 5b and Table 3). The focal points and their corresponding central positions are shown in Figure 5d.

The analysis of the simulation results confirms that the model successfully reproduces the complete ray paths. Figure 5b–d show the ray tracing results near the optical axis. In contrast to a conventional single-focus lens, the designed aspheric lens converges the incident rays into a series of discrete, predetermined axial focal points. This result visually validates the design principle of redistributing the incident laser energy among multiple axially separated foci.

To quantitatively evaluate the optical characteristics of each focus, the focal length f and corresponding effective aperture D_eff_ for each focus were substituted into the diffraction-limited spot diameter formula:

(8) $$d\;=\;2.44\;\times \;\frac{\lambda \;\times \;f}{D_{\mathrm{eff}}}$$

The calculated spot diameters for each focus are listed in Table 3. Laser-induced water breakdown in the WJGL system occurs when the laser density at the smallest spot reaches 10^10^ W/cm^2^, corresponding to an average power of 66.48 W incident on the corresponding focus. Therefore, with the proposed aspherical lens, the total laser power required to trigger breakdown across the system is 398.88 W (6 × 66.48 W). Therefore, when the total laser power is less than 398.88 W, the peak laser energy density at each focal spot in the water-jet guided laser machining system based on the six-focus aspheric lens remains below the theoretical breakdown threshold of water 10^10^ W/cm^2^, thereby avoiding the occurrence of laser-induced water breakdown. At this stage, the peak laser power densities sustained by focus 1 through focus 6 are 1.00 × 10^10^, 7.90 × 10^9^, 6.13 × 10^9^, 5.05 × 10^9^, 4.08 × 10^9^, 3.22 × 10^9^ W/cm^2^.

The simulation results confirm that the lens successfully distributes the total laser power to six discrete focal points rather than concentrating it at a single location. Consequently, the laser peak power density at any point along the beam path is significantly reduced. This increases the maximum allowable total laser power for safe operation from 66.48 W (single-focus system) to 398.88 W (The share of laser power density allocated to each focal spot is 1/6). This six-fold increase arises from the effective distribution of total power, which suppresses the local power density at each focus below the water breakdown threshold. These results demonstrate the feasibility and advantage of the aspheric lens in mitigating water breakdown in WJGL systems through spatial redistribution of the incident laser energy.

In this paper, a concept and design methodology for suppressing optical breakdown in water-jet guided laser processing by distributing laser energy via an axial multi-focal aspheric lens is proposed. The six-focal aspheric lens designed in this section is therefore only an example to demonstrate the feasibility of the proposed method. Based on this methodology, lenses with different sizes and focal lengths can be designed, or dedicated lenses can be redesigned by optimizing the aspheric surface or introducing wavefront modulation. Furthermore, the axial multi-focus aspheric lens proposed in this study has certain limitations. First, the laser energy distribution among the focal points is not perfectly uniform. Second, the alignment sensitivity of the laser water jet coupling is increased. These issues can be further addressed in future work by adopting freeform lenses or introducing a piezoelectric ceramic-driven micro-positioning stage.

## 5. Experimental Results and Discussion

### 5.1. Experiment

To quantitatively evaluate the effectiveness of the aspheric lens designed in Section 3 in enhancing water-jet guided laser machining performance, comparative microgroove cutting experiments were conducted on NiTi alloy. NiTi alloy was selected for two primary reasons. First, it has broad application potential in medical devices, aerospace, and micro-electromechanical systems (MEMS) [54,55]. Second, its high hardness, thermal sensitivity, and susceptibility to thermally induced phase transformations and crack propagation during machining [56,57] make it a representative material for assessing the high-quality, low-thermal-damage machining capability of the water-jet guided laser (WJGL) system.

**Figure 6:**
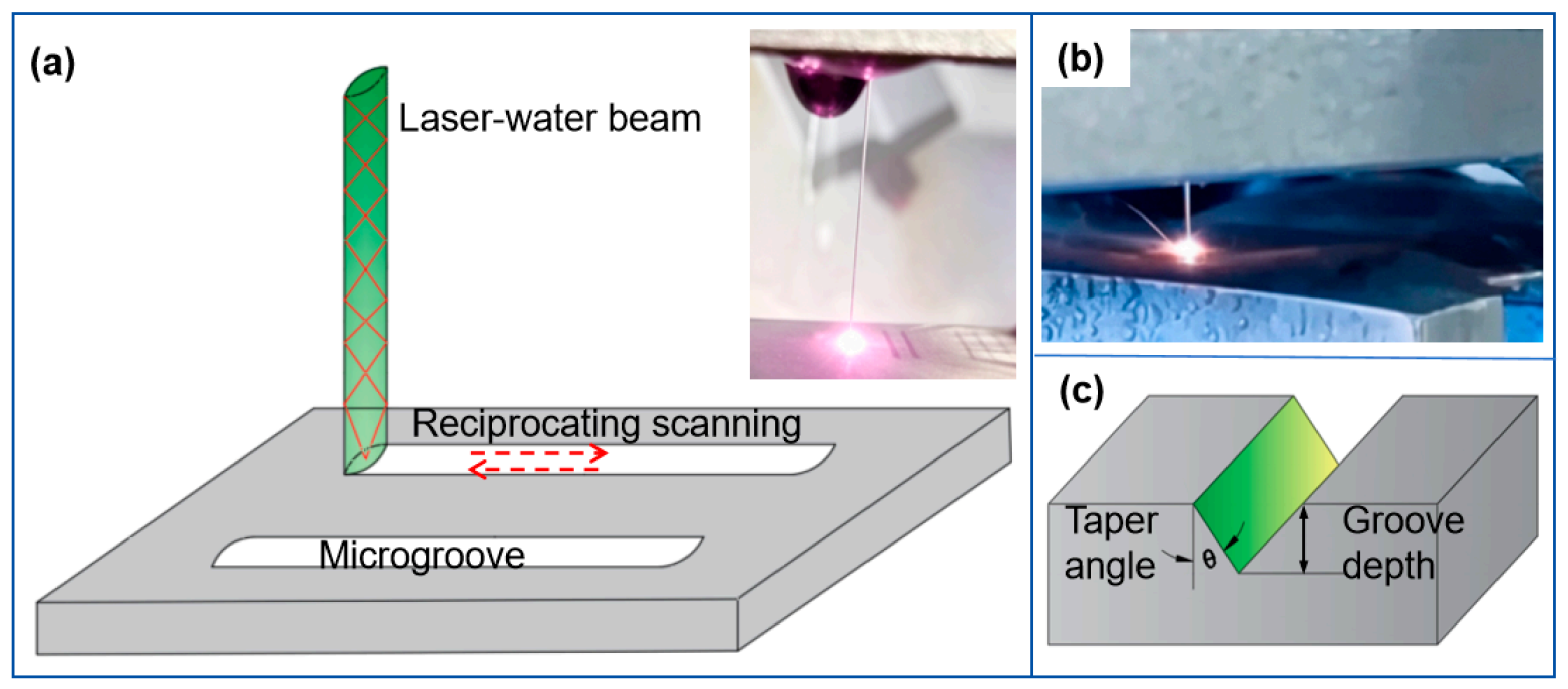
Water-jet guided laser machining path and quality indicator detection method: (**a**) machining path; (**b**) water-jet guided laser machining system; (**c**) microgroove characterization parameter measurement method.

The workpiece material was a 50 mm × 50 mm × 0.5 mm NiTi alloy plate. Its chemical composition and physical properties are listed in Table 4 and Table 5, respectively. Workpieces were ultrasonically cleaned before and after experiments in a YL1630-4080100 ultrasonic cleaner (Yunyisonic Shenzhen Co., Ltd., Shenzhen, China) with anhydrous ethanol at 50 °C (high-frequency, 80 kHz mode) for 20 min. They were then dried using an Elmadry TD30 unit (Elma Electronic GmbH, Konstanz, Germany). Groove cutting experiments were performed using a custom-built water-jet guided laser machining system (Figure 6b). Based on preliminary exploratory experiments and findings reported in the literature [58,59,60], the experimental parameters in this study were selected. The specific parameters are presented in Table 6. The machining path design is illustrated in Figure 6a. An Olympus DSX1000 3D digital microscope (Olympus Corporation, Tokyo, Japan) was employed to characterize the surface morphology and key dimensions of the machined microgrooves; the specific measurement parameters and methods are shown in Figure 6c.

### 5.2. Results and Discussion

**Figure 7:**
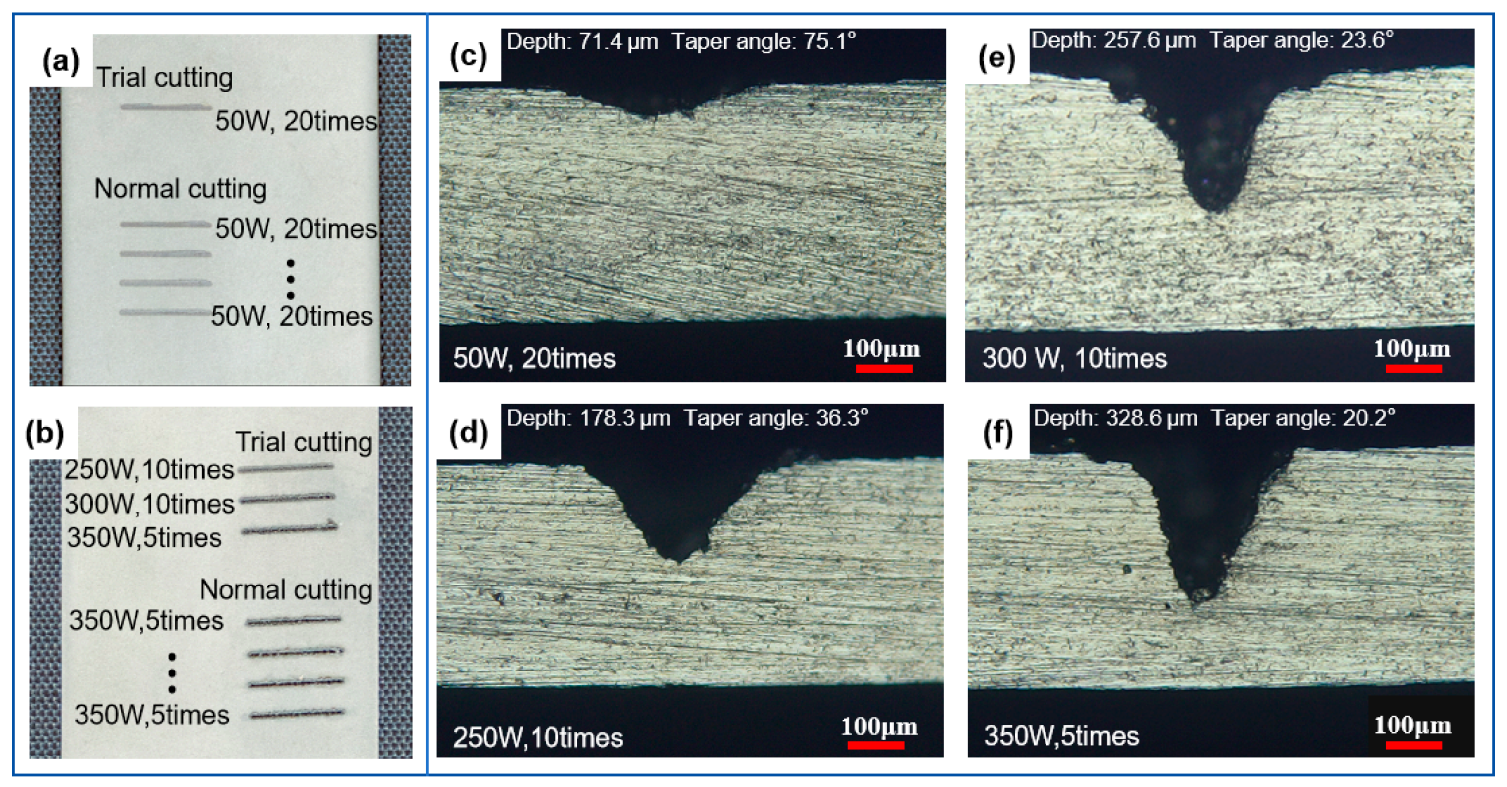
Machining results of the water-jet guided laser system based on a conventional single-focus lens and a six-focus aspheric lens, and microgroove morphologies under different parameters: (**a**) machining result using the water-jet guided laser system with a conventional single-focus lens; (**b**) machining result using the water-jet guided laser system with a six-focus aspheric lens; (**c**) microgroove morphology obtained with the conventional single-focus lens at 50 W with 20 times; (**d**) microgroove morphology obtained with the six-focus aspheric lens at 250 W with 10 times; (**e**) microgroove morphology obtained with the six-focus aspheric lens at 300 W with 10 times; (**f**) microgroove morphology obtained with the six-focus aspheric lens at 350 W with 5 times.

Microgrooves were machined on a NiTi alloy plate using water-jet guided laser processing systems based on two types of lenses. The macroscopic morphologies of the microgrooves are shown in Figure 7a,b, and the corresponding cross-sectional profiles are presented in Figure 7c–f. When using the conventional spherical single-focus lens, the laser power was limited to 50 W (safe operating power) to prevent laser-induced water breakdown. After 20 passes, a groove depth of 71.4 µm with a taper angle of 75.1° was obtained. Minor scratches can be observed on the workpiece surface in Figure 7a.

In contrast, the water-jet guided laser processing system based on the six-focus aspheric lens enabled the machining of deeper grooves with higher speed and fewer passes. As shown in Figure 7b, distinct machining marks are visible on the workpiece surface. Figure 7d–f reveal that the groove depths increased significantly with the six-focus aspheric lens-based system. Under cutting conditions of 250 W/10 times and 300 W/10 times, groove depths of 178.3 µm and 257.6 µm were achieved, respectively, as shown in Figure 7d,e. When the laser power was increased to 350 W, a groove depth of 328.6 µm was obtained after only five passes, with the taper angle reduced to 20.2°, and the corresponding groove morphology is presented in Figure 7f.

The experimental results indicate that the water-jet guided laser processing system based on the six-focus aspheric lens achieved a groove depth approximately 3.5 times that of the conventional system operating at 50 W (350 W vs. 50 W), while the taper angle was approximately 2.7 times smaller. Furthermore, Figure 7c–f show well-defined groove cross-sectional edges with no apparent heat-affected zone, confirming the inherent cooling advantage of the water jet in water-jet guided laser machining.

The notable improvement in performance is fundamentally attributed to the unique multi-focal energy redistribution capability of the aspheric lens. Its design redistributes the total laser energy into a sequence of discrete focal points along the optical axis, thereby significantly reducing the peak power density at any single focus. This mechanism effectively suppresses laser-induced water breakdown, enabling stable system operation at a substantially higher average laser power. The increased laser power directly enhances the material removal rate, resulting in a greater groove depth. Furthermore, the more uniform axial energy distribution promotes consistent material removal from the groove top to the bottom, effectively suppressing the melting and widening of the groove entrance caused by excessive energy concentration. Consequently, the water-jet guided laser machining system based on the axial multi-focus aspheric lens is capable of producing microgrooves with steeper sidewalls and a more uniform profile.

Furthermore, the experiments designed in this section are intended to demonstrate the practicality of the lens designed in Section 4, rather than being process parameter optimization experiments. In future work, experimental studies on the influence of key parameters such as laser pulse width and pulse frequency on system performance can be further conducted, and the applicability of the current optical design scheme under different operating conditions can be investigated.

## 6. Conclusions

This study successfully designed and validated an aspheric multi-focal lens for water-jet guided laser machining, effectively alleviating the power limitation imposed by laser-induced water breakdown. The research results are summarized as follows:

1. The designed aspheric lens transforms a single focal point into multiple focal points distributed along the optical axis, distributing the laser energy originally concentrated at a single focus among multiple foci and significantly reducing the peak power density at each focus. The six-focus aspheric lens designed in this work increases the usable laser power of the system from 63.6 W to 398.88 W, an increase of 335.28 W, laying a foundation for high-power stable water-jet guided laser machining.
2. The system based on the six-focus aspheric lens operated at 350 W during machining, compared to only 50 W for the system based on the spherical lens, representing an increase of 300 W in usable power.
3. In comparative NiTi alloy microgroove cutting experiments, the system employing the six-focus aspheric lens achieved an approximately 3.5-fold increase in groove depth (from 71.4 µm to 328.6 µm) and a 2.7-fold reduction in taper angle (from 75.1° to 20.2°).

Based on the water-jet guided laser machining experimental platform employing the aspheric lens optical system, the processing performance has been significantly enhanced, which is fundamentally attributed to the unique multi-focus energy redistribution capability of the aspheric lens. This study thus provides an effective power enhancement solution for water-jet guided laser technology. The core multi-focal beam shaping concept offers broad application prospects in laser processing, particularly for the high-quality manufacturing of difficult-to-machine materials such as thick-walled composites and hard brittle materials, by ensuring simultaneous optimization of machining efficiency and quality through stable coupling and axially homogenized energy distribution.

## Figures and Tables

**Table 1:** Main parameters of the water-jet guided laser processing system.

Module	Parameter	Value
Laser	Laser wavelength	1064 nm
	Oscillation mode	CW/SQ
	Laser power	≤1.5 kW
	Laser frequency	≤50 kHz
High-pressure water supply system	Water pressure	0–30 MPa
	Nozzle diameter	0.1–0.5 mm
CNC machine tool	Number of axes	3-axis
	Maximum travel	500 mm × 300 mm × 300 mm
	Maximum feed rate	1000 mm/s
	Repeatability	±3 µm

**Table 2:** Parameters of the optical path system and aspheric lens.

Optical Path System Parameters	Value	Aspheric Lens Parameters	Value
Lens material and refractive index	BK7, n = 1.5168	c	0.0195
Laser incidence angle/°	≥48.75°	k	−1
Lens rear surface radius/mm	15	A_4_	3.42 × 10^−6^
Lens focal length range/mm	99–101	A_6_	−2.58 × 10^−8^
Nozzle parameters r × h/mm	0.25 × 1	A_8_	4.88 × 10^−10^
Calculation step size/mm	0.02	A_10_	−1.89 × 10^−12^

**Table 3:** Focus point parameters.

Focus Number	Focal Length/mm	Input Beam Diameter Range/mm	Spot Diameter/µm
1	99.6	12.5–14.0	18.4
2	99.8	11.0–12.5	20.7
3	100	10.0–11.0	23.5
4	100.2	9.0–10.0	25.9
5	100.4	8.0–9.0	28.8
6	100.6	0–8.0	32.4
0	–	14.0–15.0	Aperture margin

**Table 4:** Chemical composition of NiTi alloy sheet (ω, %).

Element	Ni	C	Co	Cu	Cr	H	Fe	Nb	N + O	Ti
Value	55.75	0.005	0.005	0.005	0.005	0.001	0.005	<0.025	<0.039	Bal.
Standard	54.5–57.0	≤0.050	≤0.050	≤0.010	≤0.010	≤0.050	≤0.050	≤0.025	≤0.050	Bal.

**Table 5:** Physical properties of NiTi alloy sheet.

Physical Property	Melting Point/°C	Density/kg/m^3^	Poisson’s Ratio/ν	Tensile Strength/MPa	Elongation/%	Grain Size
Value	1310	6450	0.33	706	15	8.5

**Table 6:** Experimental parameters.

Experimental Parameters	Single-Focus Spherical Lens	Six-Focus Aspheric Lens
Nozzle diameters	0.5 mm	0.5 mm
Laser power	50 W	250/300/350 W
Number of processing	20 times	10/10/5 times
Water beam pressure	2.2 MPa	2.2 MPa
Cutting speed	0.5 mm/s	0.5 mm/s

## Data Availability

The datasets used or analyzed during the current study are available from the corresponding author on reasonable request.

## Appendix A. Derivation of Equation (4)

Consider a collimated incident beam, as illustrated in Figure 3. The model is based on three key assumptions: Refraction occurs once at the front surface (aspherical) and once at the rear surface (planar) of the lens. The lens material is a homogeneous, isotropic medium with a refractive index n (relative to vacuum). The media on the left and right sides of the lens are vacuum and air, with refractive indices n_1_ = 1 and n_2_ ≈ 1, respectively. Owing to the rotational symmetry of the system, only the meridional cross-section is considered.

Let the coordinates of an arbitrary point on the curved surface be P(z_i_, r_i_), and those of a point on the planar surface be Q(OT, r). Define tanα = k. Let F_1_ and F_2_ denote the axial coordinates of the foci for the on-axis and marginal rays, respectively, with f_1_ > f_2_, and let R be the maximum radius of the planar surface.

Based on the above conditions, the derivation of Equation (4) is as follows:

(1) Snell’s law applied at points P and Q, respectively:

(A1) $$n_{1}\sin\alpha =n\sin\beta$$

(A2) $$n\sin\left(\alpha -\beta \right)=n_{2}\sin\gamma$$

With $n_{2}\;\approx \;n_{1}\;=\;1$, we obtain

(A3) sinα=nsinβ

(A4) $$n\sin\left(\alpha -\beta \right)=\;\sin\gamma$$

(2) From the assumption

(A5)
tanα=k

It follows that

(A6) $$\sin\alpha =\frac{k}{\sqrt{k^{2}+1}}$$

(3) Substituting Equation (A6) into Equation (A3) gives

(A7)
$$\sin\beta =\frac{k}{n\sqrt{k^{2}+1}}$$

Equation (A7) leads to

(A8) $$\tan\beta =\frac{k}{\sqrt{n^{2}k^{2}+{\;n}^{2}-k^{2}}}$$

(4) Using the trigonometric identity $\sin\left(\alpha -\beta \right)\;=\;\sin\alpha \cos\beta -\cos\alpha \sin\beta$, we obtain

(A9)
$$\sin\left(\alpha -\beta \right)=\frac{\left(\sqrt{n^{2}k^{2}+{\;n}^{2}-k^{2}}-1\right)k}{n\left(k^{2}+1\right)}$$

Consequently,

(A10) $$\tan\left(\alpha -\beta \right)=\frac{\left(\sqrt{n^{2}k^{2}+{\;n}^{2}-k^{2}}-1\right)k}{\sqrt{n^{2}k^{2}+{\;n}^{2}-k^{2}}+k^{2}}$$

(5) From Equation (A10), the expression for the refracted segment PQ is derived as

(A11)
$$r-r_{i}=\frac{\left(\sqrt{n^{2}k^{2}+n^{2}-k^{2}}-1\right)k}{\sqrt{n^{2}k^{2}+{\;n}^{2}-k^{2}}+k^{2}}\left(z-z_{i}\right)$$

Since z = OT, Equation (A11) yields

(A12) $$r=r_{i}+\frac{\left(\sqrt{n^{2}k^{2}+n^{2}-k^{2}}-1\right)k}{\sqrt{n^{2}k^{2}+n^{2}-k^{2}}+k^{2}}\left(\mathrm{OT}-z_{i}\right)$$

Thus, the coordinates of point Q are $Q\;\left(\mathrm{OT},\;r_{i}\;+\;\frac{\left(\sqrt{n^{2}k^{2}\;+\;n^{2}\;-\;k^{2}}\;-\;1\right)k}{\sqrt{n^{2}k^{2}\;+\;n^{2}\;-\;k^{2}\;}+{\;k}^{2}}\left(\mathrm{OT}\;-\;z_{i}\right)\right)$.

(6) Similarly, from Equations (A4) and (A9), we obtain

(A13)
$$\sin\gamma =\frac{\left(\sqrt{n^{2}k^{2}+n^{2}-k^{2}}-1\right)k}{k^{2}+1}$$

This leads to

(A14) $$\tan\gamma =\frac{\left(\sqrt{n^{2}k^{2}+{\;n}^{2}-k^{2}}-1\right)k}{\sqrt{\left(2-n^{2}\right)k^{4}+\left(1-n^{2}\right)k^{2}+2k^{2}\sqrt{n^{2}k^{2}+n^{2}-k^{2}\;}+1}}$$

(7) Let the coordinates of point F be ($\tilde{z}$, 0). The expression for the refracted segment Qf is

(A15)
$$0-\left[r_{i}+\frac{\left(\sqrt{n^{2}k^{2}+n^{2}-k^{2}}-1\right)k}{\sqrt{n^{2}k^{2}+n^{2}-k^{2}}+k^{2}}\left(\mathrm{OT}-z_{i}\right)\right]=\tan\gamma \left(\tilde{z}-\mathrm{OT}\right)$$

Finally, the axial coordinate of point f is given by

(A16) $$\tilde{z}=\mathrm{OT}-\left[r_{i}+\frac{\left(\sqrt{n^{2}k^{2}+n^{2}-k^{2}}-1\right)k}{\sqrt{n^{2}k^{2}+n^{2}-k^{2}}+k^{2}}\left(\mathrm{OT}-z_{i}\right)\right]\;\times \;\frac{\sqrt{\left(2-n^{2}\right)k^{4}+\left(1-n^{2}\right)k^{2}+2k^{2}\sqrt{n^{2}k^{2}+n^{2}-k^{2}}+1}}{\left(\sqrt{n^{2}k^{2}+n^{2}-k^{2}}-1\right)k}$$
